# The Effects of Acute Cognitively Engaging Physical Activity on Executive Function in Preschool Children: Evidence from Behavioral and fNIRS Measures

**DOI:** 10.3390/bs15121712

**Published:** 2025-12-10

**Authors:** Anlong Du, Ke Ning, Chunzi Shangguan, Chen Wang, Bingjun Wan, Aiping Chi

**Affiliations:** 1 School of Physical Education, Jiangsu Normal University, Xuzhou 221116, China; 6020250049@jsnu.edu.cn; 2 School of Physical Education, Shaanxi Normal University, Xi’an 710119, China; 3 Shenzhen Longhua School Affiliated to East China Normal University, Shenzhen 518109, China

**Keywords:** cognitively engaging physical activity, executive function, preschool children, functional near-infrared spectroscopy

## Abstract

Executive function is crucial for the physical and mental health as well as social adaptation of preschool children, and cognitively engaging physical activity may serve as an effective intervention. This study employed a pre-post experimental design with a repeated measures ANOVA to examine the intervention effects and underlying mechanisms of a 20 min cognitively engaging physical activity on preschool children’s executive function. A total of 56 preschool children were recruited and randomly assigned to either the cognitively engaging physical activity group or the conventional physical activity group. Executive function was assessed before and after the intervention using Go/No-Go, 1-back, and dimension-changing card classification tasks. Functional near-infrared spectroscopy was employed to monitor changes in oxygenated hemoglobin concentration in the prefrontal cortex during cognitive tasks. Results indicate that acute cognitively engaging physical activity effectively modulates oxygenated hemoglobin concentration in specific regions of the prefrontal cortex in preschool children, leading to an immediate enhancement in working memory capacity. This approach demonstrates potential advantages in inhibitory control, while no significant differences were observed in cognitive flexibility. Furthermore, post-intervention changes in inhibitory control and working memory showed significant positive correlations with changes in prefrontal oxygenated hemoglobin concentration. These findings provide scientific evidence for applying cognitive engagement elements in cognitive development and theoretical support for designing targeted physical activity interventions.

## 1. Introduction

Executive function (EF) refers to a set of cognitive skills necessary for individuals to consciously regulate their thoughts, behaviors, and emotions to achieve specific goals (Zelazo et al., 2016). It comprises three subcomponents: inhibitory control (IC), working memory (WM), and cognitive flexibility (CF) (Lehto et al., 2003; Miyake et al., 2000). IC refers to an individual’s ability to demonstrate self-control, engage in more adaptive behaviors, and avoid impulsive responses (Tominey & McClelland, 2016). WM denotes an individual’s capacity to retain and select relevant information while manipulating it (Baddeley, 2010). CF signifies an individual’s ability to shift perspectives or consider multiple viewpoints when necessary (Dajani & Uddin, 2015). These psychological skills promote the organization and control of goal-directed behavior (Best, 2010), jointly influencing an individual’s attention, emotional, and physiological responses to stimuli (Blair & Raver, 2014), thereby supporting more effective learning (Diamond, 2013). Research indicates that the development of EFs during the preschool years correlates with language and mathematical abilities (Blair & Razza, 2007; Bull et al., 2008; Diamond & Lee, 2011; Verdine et al., 2014), long-term physical and mental health (Diamond & Lee, 2011), and positive social relationships (Diamond, 2012). Early EF development provides children with significant advantages at school entry (Blair & Razza, 2007), promotes subsequent academic achievement (Raver et al., 2011), and yields more pronounced effects among disadvantaged groups with socio-economic disparities (Fitzpatrick et al., 2014).

Physical activity, as an economical, accessible, and child-friendly educational intervention, has been widely applied in the field of cognitive development (Carson et al., 2016; J.-Y. Zhang et al., 2022). Numerous studies reveal that physical activity positively influences cognitive abilities, including executive functions (Pindus et al., 2016). However, not all physical activities yield equivalent cognitive benefits (Tomporowski et al., 2015). Differences in exercise intensity, intervention duration, and activity type result in varying effects on executive function improvement (J. Li et al., 2025; O’Callaghan et al., 2024). Regarding exercise intensity, moderate-intensity acute physical activity significantly enhances children’s executive function more effectively than other intensities (Ji et al., 2019). Moreover, the relationship between cognitive performance and exercise intensity after acute physical activity follows an inverted U-shaped curve (Kamijo et al., 2004). Regarding intervention duration, both acute and long-term physical activity effectively enhance EF in children and adolescents (Liu et al., 2020). With respect to potential mechanisms, acute physical activity may enhance EF by increasing physiological arousal and optimizing the allocation of cognitive resources (Tomporowski, 2003), by transiently elevating catecholaminergic neurotransmitters such as dopamine and epinephrine (McMorris, 2016), and by upregulating brain-derived neurotrophic factor in central and peripheral circulation (Loprinzi et al., 2013). Furthermore, a study has demonstrated that acute physical activity lasting 20 min yields significantly greater cognitive benefits than 10 min or 45 min sessions (Chang et al., 2015). Concerning exercise modality, the level of cognitive engagement incorporated into physical activity has garnered extensive attention (Tomporowski et al., 2015). Appropriate cognitive stimulation can enhance EF by modulating physiological and psychological arousal levels (Pesce, 2012). Physical activities incorporating cognitive engagement elements offer greater advantages than simple repetitive activities, yielding additional cognitive benefits (Preston et al., 2025). Cognitively engaging physical activity (CEPA) incorporates conditions such as situational interference, mental control, and exploration/discovery into physical activities. It requires participants to allocate attention resources and cognitive effort appropriately within challenging and complex environments (Kolovelonis et al., 2022b). As a form of physical activity intervention, it is characterized by its operationalizability of cognitive engagement and high interest in physical activity contexts, making it particularly suitable for preschool children. Multiple studies indicate that CEPA effectively promotes cognitive development, attention, and EF in children and adolescents (Jia et al., 2021; Kolovelonis & Goudas, 2023; Vazou & Mavilidi, 2021). Existing research confirms that acute physical activity interventions involving cognitive demands can produce immediate positive effects on young children’s performance in motor inhibition tasks (Stein et al., 2017). Further studies indicate that short-duration physical activities with moderate cognitive engagement yield more significant improvements in EF and self-regulation compared to activities with high or low cognitive demands (Ureña et al., 2020). Although existing research confirms the effectiveness of acute physical activity and cognitive engagement in enhancing EF, evidence regarding the impact of cognitively engaged physical activity on preschool children’s EF remains limited and inconclusive. Compared to conventional physical activity, the intervention effects and underlying mechanisms of such activities require further clarification.

The prefrontal cortex (PFC) plays a central role in cognitive control and EF. Particularly during the cognitive development of children and adolescents, the maturation of its structure and function is closely associated with improvements in cognitive abilities (Casey et al., 2018; Friedman & Robbins, 2022; Luna et al., 2015). Preschool children exhibit significant activation in prefrontal regions during executive function tasks (Moriguchi & Hiraki, 2013). As a higher-order association cortex within the frontal lobe, the dorsolateral prefrontal cortex (DLPFC) occupies the anterior portion of the middle and superior frontal gyri, adjacent to the posterior part of the precentral gyrus. It corresponds roughly to Brodmann areas 9 and 46 and is mainly responsible for maintaining and updating WM, controlling attention, monitoring conflicts, and carrying out goal-directed behaviors. It is recognized as one of the core structures within the cognitive control system (Miller & Cohen, 2001; Niendam et al., 2012). The frontopolar cortex (FPC) is the most anterior region of the PFC, located at the foremost position of the frontal lobe within Brodmann area 10. It plays a pivotal role in regulating higher-order cognitive processes (Friedman & Robbins, 2022).

Currently, functional near-infrared spectroscopy (fNIRS) serves as a non-invasive brain imaging technique. By attaching optical probes to the tissue surface to emit and receive near-infrared light (650–1000 nm), it measures concentration changes in oxyhemoglobin (HbO_2_) and deoxyhemoglobin (HHb), thereby reflecting hemodynamic alterations in the tissue. This method is commonly employed to examine cortical activation during cognitive tasks and infer underlying neural activity in the brain (Herold et al., 2018; Huppert et al., 2006). Its portability and low sensitivity to motion artifacts make it suitable for preschool children studies (Zhan et al., 2024). Research has confirmed that acute physical activity activates physiological arousal systems, enhancing prefrontal cortical activation associated with cognitive tasks and thereby improving EF (Byun et al., 2014). Different levels of cognitive engagement also exert distinct effects on cortical neural activity. Compared to monotonous movements, physical activities involving moderate physical or cognitive demands more effectively elevate oxygenated hemoglobin concentration in the PFC (Naito et al., 2024). Currently, fNIRS technology is widely applied to monitor prefrontal activation states and analyze the mechanisms underlying effects before and after interventions, effectively elucidating the benefits of different intervention approaches on cognitive performance enhancement and their corresponding neural activity characteristics. However, the mechanisms underlying changes in cerebral blood flow within the PFC during executive function tasks in preschool children following acute cognitively engaged physical activity interventions remain unclear. Therefore, this study employs fNIRS to compare pre- and post-intervention activation patterns in the PFC. It aims to deepen understanding of the neural mechanisms underlying acute cognitive engagement during physical activity interventions that enhance preschoolers’ EF performance. This research will enrich the theoretical foundation in this field, broaden perspectives on improving preschoolers’ cognitive performance, and provide scientific evidence and practical pathways for designing and implementing cognitively engaging physical activity intervention programs.

In conclusion, cognitively engaged physical activity has surfaced as a promising intervention for preserving and augmenting individual cognitive function. However, the relationship between such activities and EF benefits, along with their underlying mechanisms, remains unclear. This study employs fNIRS technology to investigate the behavioral benefits of EF improvement before and after physical activity interventions with varying levels of cognitive engagement, as well as the characteristic changes in oxygenated hemoglobin concentration in the PFC. Specifically, this study examines (1) whether cognitively engaged physical activity yields greater improvements in EF compared to conventional physical activity among preschool children; (2) the patterns and differences in changes in PFC HbO_2_ concentration in preschool children before and after conventional and cognitively engaged physical activity interventions; and (3) the relationship between changes in behavioral measures of EF and cerebral blood flow in preschool children before and after physical activity interventions.

## 2. Materials and Methods

### 2.1. Trial Design

This study employed a parallel group design using a 2 (group) × 2 (test time) mixed-design experimental setup to investigate the acute effects of cognitive engagement factors on EF. All participating children were required to visit the experimental site twice: the first visit involved basic information collection, testing, and learning the intervention content; the second visit involved completing the physical activity intervention and data collection.

### 2.2. Procedures

#### 2.2.1. Testing Sites

The subjects for this experiment were drawn from two kindergartens under the same educational group in Xi’an City, Shaanxi Province, China. Both kindergartens were comparable in terms of geographical location, facility scale, teacher qualifications, and curriculum design. The experimental intervention sites were established in open physical activity areas within two kindergartens, featuring similar environmental conditions in terms of size and layout. To meet fNIRS data collection requirements as closely as possible, the data acquisition site was set up in a quiet, dimly lit, independent room within the kindergarten.

#### 2.2.2. Eligibility Criteria

A total of 64 preschool children were selected based on inclusion criteria: children in the senior kindergarten class, right-handedness, normal vision or corrected vision, normal intellectual development, no mental disorders, and no health conditions affecting participation in physical activities. All intervention implementers were master’s degree candidates from the School of Physical Education at Shaanxi Normal University. They received at least one day of formal training provided by the experiment designers and conducted one simulated intervention prior to the formal experiment.

#### 2.2.3. Randomization

The randomization list was prepared by a researcher who was not involved in data collection or intervention implementation, and the group assignments were implemented after the first visit. Stratified random sampling with proportional allocation was conducted at the kindergarten level to recruit participants, ensuring that the number of children from each kindergarten was proportional to its size. Then, all 64 eligible participants were randomly assigned to either the cognitively engaging physical activity group (n = 32) or the conventional physical activity group (n = 32) using the RANDBETWEEN(1,2) function in Microsoft Excel to generate random allocation codes.

#### 2.2.4. Ethical Considerations

The Academic Committee of Shaanxi Normal University has authorized this study (Scientific Ethics Review Number: 202416060). All parents or legal guardians comprehended the research protocols and provided written informed consent prior to their children’s involvement. Moreover, the testing procedure meticulously adhered to the personal choices of the participating children, who retained the autonomy to resign from the study at any moment.

### 2.3. Intervention and Comparator

The physical activity intervention in this study was of moderate intensity, defined as 64% to 95% of maximum heart rate (HRmax). The formula for calculating maximum heart rate is 220 minus age. During the experiment, children’s heart rates were monitored using the Domor portable heart rate monitor (American College of Sports Medicine, 2013). Each child received the intervention individually and completed the tasks at each station in sequence for three cycles, with each intervention session lasting 20 min. To control for teaching style effects, the intervention was delivered in a rotating stations format, with teachers providing only necessary prompts based on game rules. The balance beams, mats, toddler soccer balls, sandbags, horizontal bars, and other materials required for the intervention are all standard physical education equipment commonly used in kindergartens. To control for potential interference from the kindergarten curriculum on intervention effectiveness, all participants completed the tests between 9:30 AM and 11:30 AM.

Cognitively engaging physical activity involves elements such as situational interference, mental control, and discovery. Participating children were required to complete games based on fundamental motor skills and engage in physical activity within the game’s rules. The balance and stability motor skills segment required children to mimic static poses of different animals, locate and sequentially walk across balance beams of varying colors, and perform forward rolls toward directions randomly indicated by the instructor. The operational motor skills segment required children to dribble a ball with their feet while navigating around obstacles and throw soft beanbags toward a grid 3 m away, aiming to form a straight line. Mobility motor skills involved sideways crawling and stepping over bars of varying heights to reach the finish line; displaying red, yellow, and green signs during running to indicate stop, slow down, and normal running, respectively; and performing frog jumps in randomly indicated directions. Additional rules during the intervention required children to innovate movements and methods during play, respond promptly to unpredictable situations, and continuously adjust behavioral strategies.

Conventional physical activity similarly employed moderate-intensity activities incorporating basic motor skills, but communication with children involved only simple encouragement and instructions, excluding game rules with cognitive engagement elements. The balance and stability motor skills section required children to perform single- and double-leg static stands, balance beam walking, and forward rolls on triangular mats. The operational motor skills section required children to dribble in a straight line and throw soft beanbags at distant targets. The locomotor motor skills segment required children to perform lateral walking, running at a constant speed around the field, and forward frog jumps. Compared to cognitively engaged physical activity, rules involving cognitive participation were reduced, but identical equipment specifications and consistent station sequences were maintained. The intervention content is detailed in Table 1.

### 2.4. Outcomes

**Figure 1:**
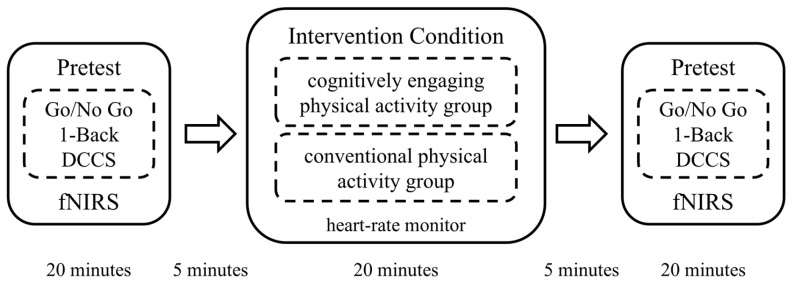
Experimental procedure diagram.

Prior to the intervention, each participant completed three EF cognitive tasks in a consistent order while concurrently undergoing fNIRS data collection. Subsequently, participants individually engaged in either a 20 min cognitively engaging physical activity intervention or a conventional physical activity intervention, with real-time heart rate monitored during the activity using a Domor portable heart rate monitor. After the exercise session, participants repeated the EF cognitive task tests and fNIRS data collection in the same order as the pre-test. Interveners are responsible only for their assigned group’s testing, with no interference between groups. Test administrators were unaware of participants’ intervention group assignments. The experimental procedure is illustrated in Figure 1.

#### 2.4.1. Executive Function

All EF tests were programmed using the PsychToolBox (PTB) toolkit in MATLAB. IC was assessed using a Go/No-Go task paradigm; WM was evaluated using a 1-back task paradigm; and CF was measured using a dimensional shift card sorting task. Prior to each task, participants received training to ensure comprehension of the task rules. During the testing phase, participants were instructed to remain seated and fixate on a “+” displayed on the screen during rest periods. Responses were recorded via keyboard operation when stimuli appeared on the computer screen, including accuracy and reaction time. Task presentation and behavioral data collection were performed using E-Prime 3.0 software.

**Figure 2:**
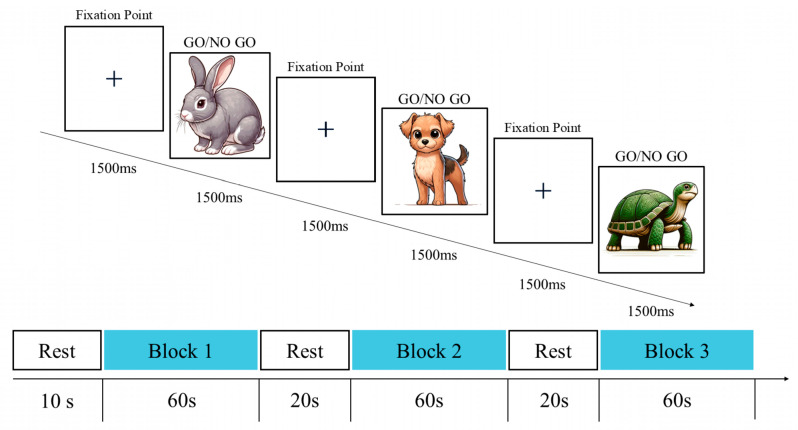
Go/No-Go Cognitive Task.

The Go/No-Go cognitive task, adapted from a classic research paradigm, is widely used to measure IC in children (Howard & Melhuish, 2017). The Go/No-Go cognitive task paradigm in this study comprised three block sets. To minimize interference from cognitive processes on response inhibition, each block set randomly distributed 10 Go trails and 10 No-Go trails (Figure 2) to assess non-selective response inhibition (Masharipov et al., 2022). Stimuli comprised animal images familiar to preschoolers. During each trial, an animal stimulus (turtle, rabbit, sheep, or dog) randomly appeared at the screen center for 1500 ms. Participants were instructed to press the spacebar immediately upon seeing any animal except the puppy (the no-go stimulus). No response was required when the puppy image appeared. To minimize potential practice effects, all subjects learned the test rules and completed 8 practice trials before the formal task. The testing procedure is illustrated in Figure 2.

**Figure 3:**
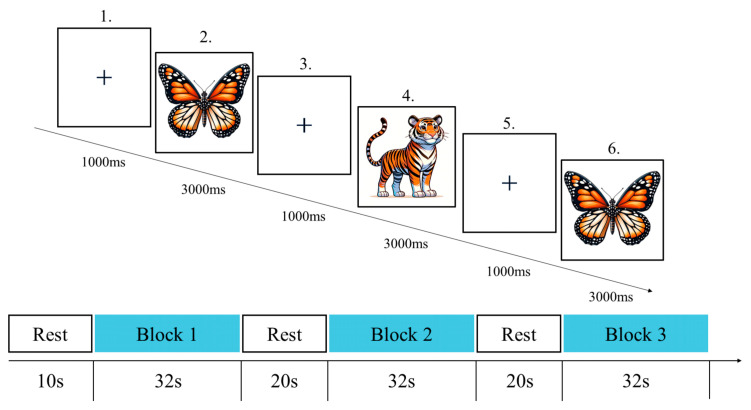
1-back Cognitive Task.

The 1-back cognitive task was adapted from the classic paradigm and modified to suit the cognitive levels of children aged 3–6 years (Chatham et al., 2011). The 1-back cognitive task paradigm in this study comprised three block sets, each containing eight trials (Figure 3). Stimuli featured five animal images familiar to children—elephant, butterfly, tiger, rabbit, and turtle—ensuring task relevance to their everyday experiences. During each trial, each animal image appeared centrally on the screen for 3000 ms, followed by a 1000-millisecond fixation point between images. Children were instructed to press the corresponding key to indicate whether the current image matched the previously displayed one, using the “F” key for match and the “J” key for no match. All trials were presented in randomized order to prevent sequence effects from influencing results. Prior to the formal task, all participants learned the test rules and completed 6 practice trials. The testing procedure is illustrated in Figure 3.

**Figure 4:**
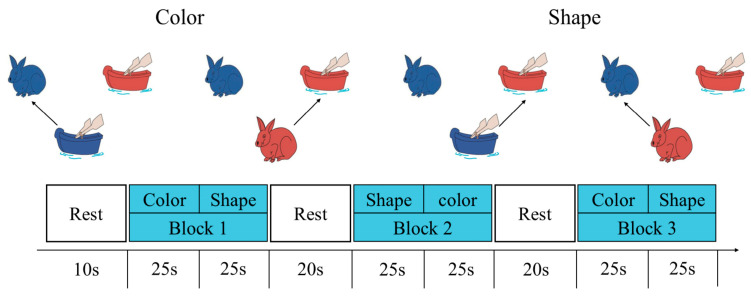
Dimensional Change Card Sorting Task.

The Dimension Change Card Sorting task has been widely used in previous studies to measure CF in preschool children (H. Li et al., 2021). The DCCS cognitive task paradigm in this study comprised three block sets, each containing 12 trials. Each block set consisted of 6 shape-matching trials and 6 color-matching trials (Figure 4). During testing, two target images (rabbit and boat) served as stimuli and were displayed in the upper half of the screen. A test image (rabbit or boat) appeared at the center bottom of the screen for 3000 ms. Participants matched the test image to the target image according to the rule: pressing the “F” key matched it to the left image, while pressing the “J” key matched it to the right image. The rule sequence for the three block sets was fixed and applied uniformly across all participants to control for learning effects. Prior to the formal task, all subjects learned the test rules and completed 6 practice trials. The testing procedure is illustrated in Figure 4.

#### 2.4.2. fNIRS

**Figure 5:**
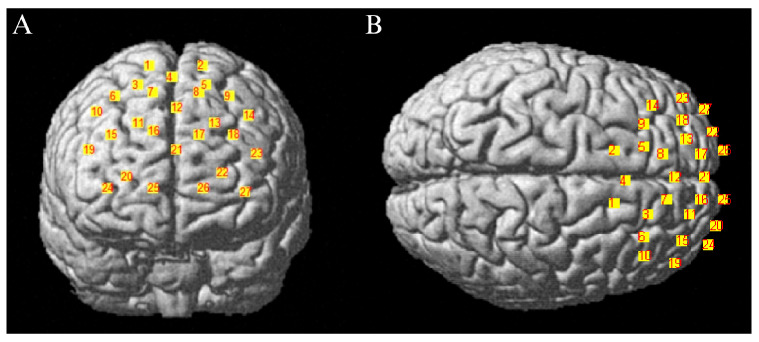
(**A**) Frontal View of the Channel Layout in Three Dimensions in Brain Regions; (**B**) Top-down Perspective of the Three-Dimensional Channel Arrangement in Brain Regions.

This study employed the Artinis Corporation (Holland) brite MKIII portable near-infrared brain imaging system to record hemodynamic changes in the PFC during subjects’ performance of WM tasks. The hardware configuration comprised 10 emitters and 8 receiver probes. Based on the distribution of brain regions associated with EF and the arrangement scheme of regions of interest (ROIs) from prior studies (Xiang et al., 2022), a 2 × 13 channel matrix layout was employed, yielding a total of 27 acquisition channels covering the PFC. Emitter and receiver probes were spaced approximately 3 cm apart, with light sources centered at wavelengths of 760/850 nm. The sampling rate was set to 25 Hz. Precise scalp coordinates for each channel were determined using the Polhemus Patriot 3D head tracking system (USA). Three-dimensional channel coordinates were acquired and spatially registered within the Matlab environment using the “NIRS_SPM” software package (https://www.nitrc.org/projects/nirs_spm/ (accessed on 15 July 2025)). Channel distribution is shown in Figure 5. Based on the spatial layout of the 27 channels, four regions of interest (ROIs) were further delineated. The correspondence between each channel and the respective brain regions is detailed in Table 2.

### 2.5. Sample Size

Sample size was determined using G*Power 3.1 to pre-set statistical power based on repeated measures ANOVA (interaction) (Faul et al., 2009). The effect size was established at a medium value of 0.25 (effect size f = 0.25), accompanied by a two-tailed α level of 0.05 and a target power of 0.95 (Bedard et al., 2021). Inputting the number of groups and measurements (n = 2), the calculated sample size for this study should exceed 54, with each group requiring at least 27 participants. After inputting the number of groups and measurements (n = 2), the calculated sample size required for this study was determined to be 54 or more. No significant differences were observed between the two groups in baseline characteristics (Table 3). During formal testing, some children were unable to complete assessments due to excessive activity and inability to remain still. Ultimately, 59 preschoolers completed all tests. During near-infrared data preprocessing, 3 children were excluded due to excessive artifacts in cerebral oxygenation signals. The final sample included 56 children: 29 males and 27 females. The groups were a cognitively engaging physical activity group (n = 27) and a conventional physical activity group (n = 29).

### 2.6. Data Processing

Using the E-Merge3 feature in E-Prime 3.0, behavioral records for all subjects were consolidated and subsequently imported into Excel for preliminary organization. Data points exceeding ±3 standard deviations from the mean were excluded. fNIRS data preprocessing was performed in the MATLAB environment (R2013b, MathWorks, Natic, MA, USA). The Oxysoft2matlab plugin provided by Artinis was used to convert raw “.oxy5” and “.oxyproj” files into “.nirs” format. Subsequently, the Homer2 toolkit was employed to convert light intensity data into hemoglobin concentration. Motion artifacts were corrected across all channels for every subject, and poor channels were flagged. A bandpass filter of 0.01–0.1 Hz was applied to suppress physiological noise such as respiration and heartbeat. Data from the two seconds preceding each block stimulus served as the baseline for correction. Blood oxygen signals from all blocks of the same stimulus type were averaged to obtain the mean values for HbO_2_, deoxygenated hemoglobin (HHb), and total hemoglobin (t-Hb). HbO_2_ exhibits a higher signal-to-noise ratio and greater sensitivity to blood oxygen changes compared to HHb. Therefore, this study employs HbO_2_ as the primary blood oxygen concentration metric for subsequent statistical analysis.

### 2.7. Statistical Methods

All data were statistically analyzed using SPSS 22.0. Data distribution characteristics were assessed using Shapiro–Wilk normality tests and Levene’s tests for homogeneity of variance. For normally distributed continuous variables, data were described as mean ± standard deviation (M ± SD). Independent samples *t*-tests were used to compare heart rate between the cognitively engaging physical activity group and the conventional physical activity group during the intervention period. Behavioral metrics and oxygenated hemoglobin concentration were analyzed by a 2 (group) × 2 (time) repeated-measures ANOVA, with time as the within-subjects variable and group as the between-subjects variable. The main analysis assessed the group × time interaction, and post hoc multiple comparisons were adjusted using Bonferroni correction for *p*-values. Pearson correlation analyses examined the linear relationship between changes in HbO_2_ concentrations across four regions of interest and changes in behavioral measures.

## 3. Results

### 3.1. Physical Activity Intensity

An independent samples *t*-test was used to analyze and compare the mean heart rates of the participating children during physical activity to assess exercise intensity. Results showed that during the intervention period, the difference in heart rates between the cognitively engaging physical activity group and the conventional physical activity group was not significant (*p* > 0.05). The mean heart rates for the two groups were 161.91 beats/min and 158.24 beats/min, respectively, indicating moderate-intensity physical activity. Mean heart rates are presented in Table 4.

### 3.2. Differences in Inhibitory Control by Activity Group

#### 3.2.1. Behavioral Results

**Figure 6:**
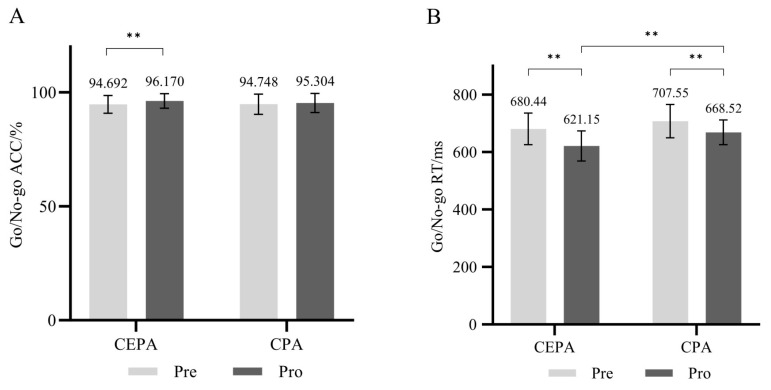
(**A**) Go/No-go Task Accuracy Rate; (**B**) Go/No-go Cognitive Task Reaction Time. ** *p* < 0.01.

For the Go/No-Go cognitive task, repeated measures ANOVA revealed a significant main effect of time on accuracy (*F* = 5.077, *p* < 0.05, $\eta_{P}^{2}$ = 0.149). Simple effects analysis revealed that under different group conditions, the post-test accuracy rate of the cognitively engaging physical activity group was significantly higher than the pre-test rate (*F* = 5.176, *p* < 0.05, $\eta_{P}^{2}$ = 0.087), while no significant difference existed between the pre- and post-test accuracy rates of the conventional physical activity group (*p* > 0.05). Across different time conditions, no significant differences existed in correct response rates between pre- and post-tests for either group (*p* > 0.05). Results are shown in Figure 6A.

The main effect of time on correct reaction time was significant (*F* = 49.550, *p* < 0.01, $\eta_{P}^{2}$ = 0.479), and the main effect of group was significant (*F* = 9.442, *p* < 0.01, $\eta_{P}^{2}$ = 0.149). The interaction effect between time and group was not significant (*p* > 0.05). Simple effects analysis revealed that, under different group conditions, the post-test correct reaction time in the cognitive engagement group was significantly lower than the pre-test (*F* = 34.794, *p* < 0.01, $\eta_{P}^{2}$ = 0.231), while the post-test correct reaction time in the conventional physical activity group was significantly lower than the pre-test (*F* = 16.195, *p* < 0.01, $\eta_{P}^{2}$ = 0.392). Under different time conditions, no significant difference existed in correct reaction times between the two groups during the pretest (*p* > 0.05). In the post-test, the correct reaction time of the cognitive engagement group was significantly lower than that of the conventional physical activity group (*F* = 13.730, *p* < 0.01, $\eta_{P}^{2}$ = 0.203). Results are shown in Figure 6B.

#### 3.2.2. fNIRS Results

**Figure 7:**
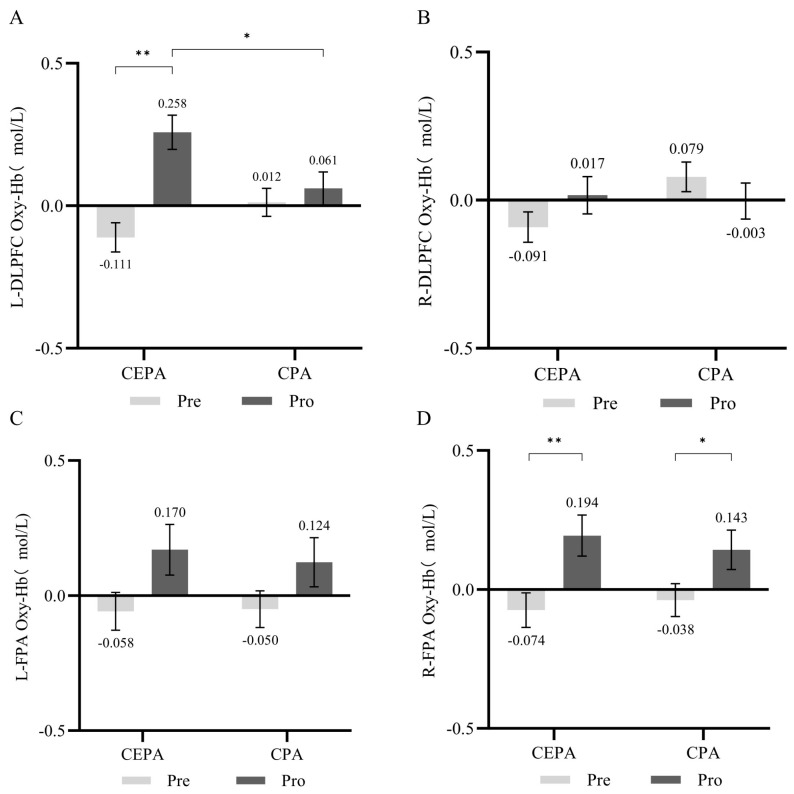
(**A**) HbO_2_ Concentration in the L-DLPFC During Go/No-Go Cognitive Tasks; (**B**) HbO_2_ Concentration in the R-DLPFC During Go/No-Go Cognitive Tasks; (**C**) HbO_2_ Concentration in the L-FPA During Go/No-Go Cognitive Tasks; (**D**) HbO_2_ Concentration in the R-FPA During Go/No-Go Cognitive Tasks. * *p* < 0.05, ** *p* < 0.01.

For fNIRS results during the Go/No-Go cognitive task, repeated-measures ANOVA revealed a significant interaction effect between time and group in the L-DLPFC (*F* = 9.436, *p* < 0.01, $\eta_{P}^{2}$ = 0.024). Simple effects analysis revealed that in the cognitively engaging physical activity group, post-test HbO_2_ concentration was significantly higher than pre-test levels across all group conditions (*F* = 24.314, *p* < 0.01, $\eta_{P}^{2}$ = 0.059). No significant differences in HbO_2_ concentration existed between groups at pre-test across different time conditions (*p* > 0.05). While at post-test, the cognitive engagement group showed significantly higher HbO_2_ levels than the conventional exercise group (*F* = 5.605, *p* < 0.05, $\eta_{P}^{2}$ = 0.014). Results are shown in Figure 7A. No significant interaction effect between time and group was observed in the R-DLPFC (*p* > 0.05). Results are shown in Figure 7B. A significant time effect was observed in the left frontal pole (*F* = 5.662, *p* < 0.05, $\eta_{P}^{2}$ = 0.033). Simple effects analysis revealed no significant differences between groups under different time conditions (*p* > 0.05). Results are shown in Figure 7C. The time effect in the right frontal pole was significant (*F* = 12.221, *p* < 0.01, $\eta_{P}^{2}$ = 0.069). Simple effects analysis revealed that under different group conditions, the cognitively engaging physical activity group exhibited significantly higher HbO_2_ concentrations at post-test compared to pre-test (*F* = 8.387, *p* < 0.01, $\eta_{P}^{2}$ = 0.048), while the conventional physical activity group also exhibited significantly higher post-test HbO_2_ concentrations than pre-test levels (*F* = 4.134, *p* < 0.05, $\eta_{P}^{2}$ = 0.024). No significant between-group differences were observed at either pre-test or post-test time points (*p* > 0.05). Results are shown in Figure 7D.

### 3.3. Differences in Working Memory by Activity Group

#### 3.3.1. Behavioral Results

**Figure 8:**
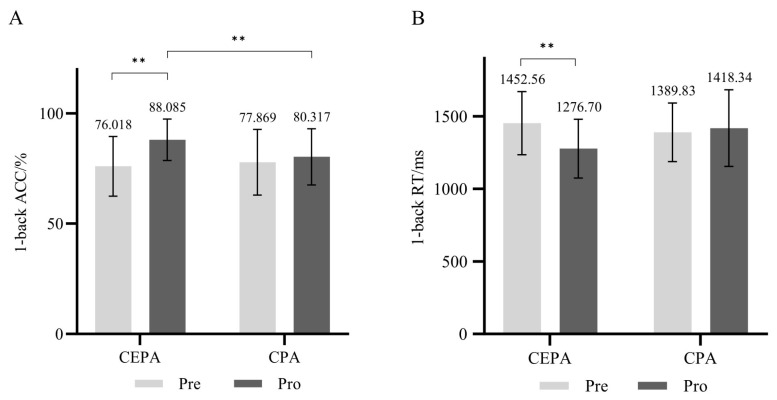
(**A**) 1-back Task Accuracy Rate; (**B**) 1-back Cognitive Task Reaction Time. ** *p* < 0.01.

For the 1-back cognitive task, the repeated measures ANOVA revealed a significant main effect of time on accuracy (*F* = 14.501, *p* < 0.01, $\eta_{P}^{2}$ = 0.212). The main effect of group was not significant (*p* > 0.05), nor was the interaction between time and group (*p* > 0.05). Simple effects analysis revealed that under different group conditions, the post-test accuracy rate of the cognitively engaging physical activity group was significantly higher than the pre-test rate (*F* = 15.922, *p* < 0.01, $\eta_{P}^{2}$ = 0.228), while no significant difference existed in the accuracy rates between pre- and post-tests for the conventional physical activity group (*p* > 0.05). Across different time conditions, no significant difference existed in pretest accuracy between the two groups (*p* > 0.05). However, at post-test, the cognitive engagement group demonstrated significantly higher accuracy than the conventional physical activity group (*F* = 4.362, *p* < 0.05, $\eta_{P}^{2}$ = 0.075). Results are shown in Figure 8A.

The main effect of time on correct reaction time was significant (*F* = 4.969, *p* < 0.05, $\eta_{P}^{2}$ = 0.084), while the main effect of group was not significant (*p* > 0.05). The interaction effect between time and group was significant (*F* = 4.913, *p* < 0.05, $\eta_{P}^{2}$ = 0.083). Simple effects analysis revealed that in the cognitive engagement group, post-test correct reaction times were significantly lower than pre-test times under different group conditions (*F* = 9.540, *p* < 0.01, $\eta_{P}^{2}$ = 0.150). No significant difference existed between pre- and post-test correct reaction times in the conventional physical activity group (*p* > 0.05). Across different time conditions, no significant differences were found in correct reaction times between pre- and post-tests for either group (*p* > 0.05). Results are shown in Figure 8B.

#### 3.3.2. fNIRS Results

**Figure 9:**
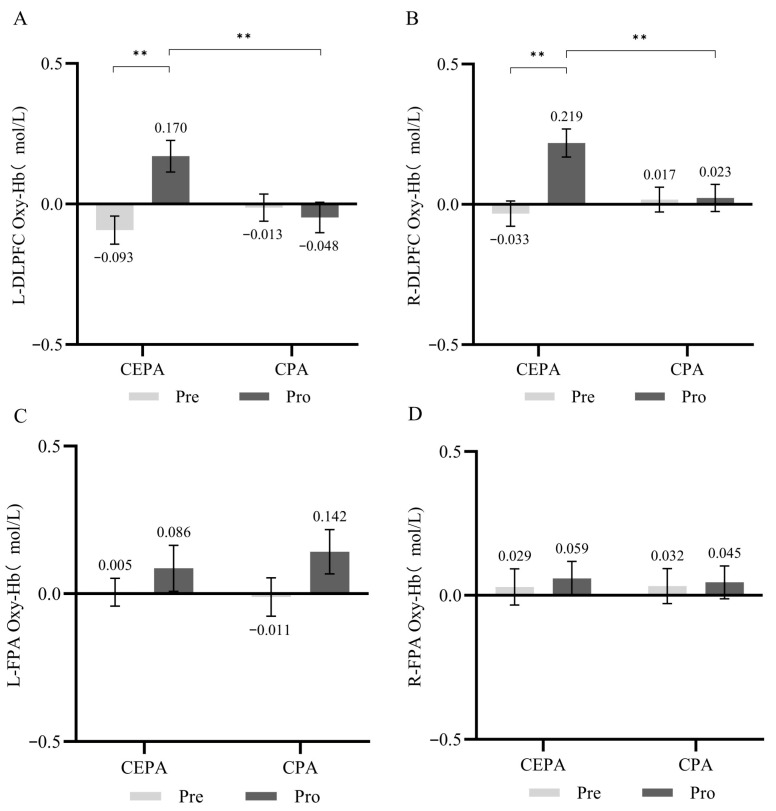
(**A**) HbO_2_ Concentration in the L-DLPFC During 1-back Cognitive Tasks; (**B**) HbO_2_ Concentration in the R-DLPFC During 1-back Cognitive Tasks; (**C**) HbO_2_ Concentration in the L-FPA During 1-back Cognitive Tasks; (**D**) HbO_2_ Concentration in the R-FPA During 1-back Cognitive Tasks. ** *p* < 0.01.

For the 1-back cognitive task, repeated measures ANOVA revealed a significant interaction effect between time and group for the L-DLPFC (*F* = 8.335, *p* < 0.01, $\eta_{P}^{2}$ = 0.021). Simple effects analysis revealed that under different group conditions, the post-test HbO_2_ concentration in the cognitively engaging physical activity group was significantly higher than the pre-test (*F* = 12.583, *p* < 0.01, $\eta_{P}^{2}$ = 0.031); no significant differences in HbO_2_ concentrations were observed between the two groups at baseline across different time conditions (*p* > 0.05); however, at post-test, the cognitively engaging physical activity group exhibited significantly higher HbO_2_ concentrations than the conventional physical activity group (*F* = 7.961, *p* < 0.01, $\eta_{P}^{2}$ = 0.020). Results are shown in Figure 9A. A significant time-by-group interaction effect was observed in the R-DLPFC (*F* = 7.560, *p* < 0.01, $\eta_{P}^{2}$ = 0.019). Simple effects analysis revealed that post-test HbO_2_ concentrations in the cognitive engagement group were significantly higher than pre-test levels under all group conditions (*F* = 15.250, *p* < 0.01, $\eta_{P}^{2}$ = 0.038). At baseline, no significant difference in HbO_2_ concentration was observed between groups (*p* > 0.05). At post-test, the cognitive engagement group showed significantly higher levels than the conventional exercise group (*F* = 7.957, *p* < 0.01, $\eta_{P}^{2}$ = 0.020). Results are shown in Figure 9B. The main effect of time, the main effect of group, and the interaction effect between time and group in the right frontal pole region were all insignificant (*p* > 0.05). Results are shown in Figure 9C. The main effect of time, the main effect of group, and the interaction effect between time and group in the left frontal pole region were all insignificant (*p* > 0.05). Results are shown in Figure 9D.

### 3.4. Differences in Cognitive Flexibility by Activity Group

#### 3.4.1. Behavioral Results

**Figure 10:**
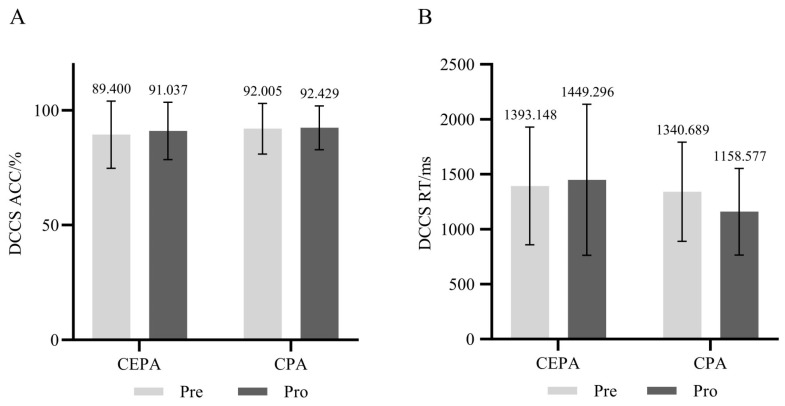
(**A**) DCCS Task Accuracy Rate; (**B**) DCCS Cognitive Task Reaction Time.

For the DCCS cognitive task, the repeated-measures ANOVA revealed that the main effect of time on accuracy, the main effect of group, and the interaction between time and group were all insignificant (*p* > 0.05). Results are shown in Figure 10A. The main effect of time on correct reaction time, the main effect of group, and the interaction between time and group were also insignificant (*p* > 0.05). Results are shown in Figure 10B.

#### 3.4.2. fNIRS Results

**Figure 11:**
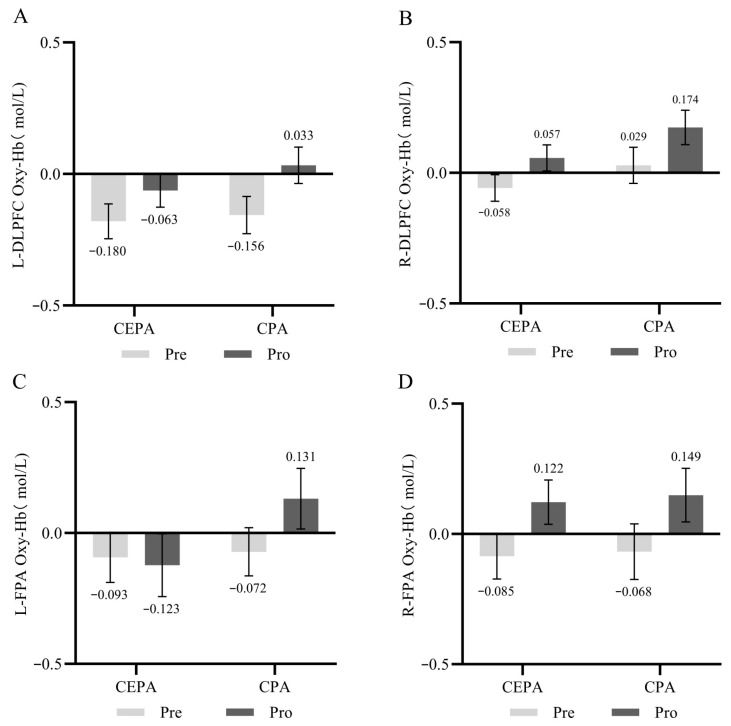
(**A**) HbO_2_ Concentration in the L-DLPFC During DCCS Cognitive Tasks; (**B**) HbO_2_ Concentration in the R-DLPFC During DCCS Cognitive Tasks; (**C**) HbO_2_ Concentration in the L-FPA During DCCS Cognitive Tasks; (**D**) HbO_2_ Concentration in the R-FPA During DCCS Cognitive Tasks.

The interaction effect between time and group in the L-DLPFC was not significant (*p* > 0.05). Results are shown in Figure 11A. The interaction effect between time and group for the R-DLPFC was not significant (*p* > 0.05). Results are shown in Figure 11B. The interaction effect between time and group for the left frontal pole was not significant (*p* > 0.05). Results are shown in Figure 11C. The interaction effect between time and group for the right frontal pole was not significant (*p* > 0.05). Results are shown in Figure 11D.

### 3.5. Correlation Between Executive Function and Cortical Activation

Pearson correlation analyses were conducted between the change in HbO_2_ concentration (post-test minus pre-test) across four regions of interest and the change in Go/No-Go accuracy and correct reaction time (post-test minus pre-test). Results showed that the change in HbO_2_ concentration in the L-DLPFC was significantly negatively correlated with the change in Go/No-Go correct reaction time (*r* = −0.501, *p* < 0.01). The change in HbO_2_ concentration in the left frontal pole showed a significant negative correlation with the change in Go/No-Go correct reaction time (*r* = −0.411, *p* < 0.01). No significant correlations were found in the remaining brain regions (*p* > 0.05). Results are presented in Table 5.

Pearson correlation analyses were conducted between HbO_2_ concentration changes (post-test minus pre-test) in four regions of interest and 1-back accuracy and correct reaction time changes (post-test minus pre-test). Results showed that HbO_2_ concentration changes in the L-DLPFC were significantly negatively correlated with 1-back correct reaction time changes (*r* = −0.299, *p* < 0.05). The change in HbO_2_ concentration in the right frontal pole showed a significant negative correlation with the change in 1-back correct reaction time (*r* = −0.278, *p* < 0.05). No significant correlations were found in the remaining brain regions (*p* > 0.05). Results are presented in Table 6.

## 4. Discussion

### 4.1. The Immediate Effects of CEPA on Inhibitory Control and Its Neural Mechanisms

The findings of this study demonstrate that, subsequent to an acute physical activity intervention, the average reaction times for correct responses in the Go/No-Go task were markedly decreased among the participating children. This finding may reflect a general effect of repeated testing or physical activity, aligning with prior research outcomes (Browne et al., 2016). Wen et al. (2021) found that after 40 min of resistance training, coordination exercises, or soccer practice, children demonstrated significantly improved performance on IC tasks. According to arousal theory, the immediate enhancement of executive function following acute physical activity may stem from the elevated physiological and psychological arousal levels it induces. This, in turn, optimizes the allocation of attentional resources, improves information processing efficiency, and ultimately enhances performance on IC tasks (B. Zhang et al., 2020). Concurrently, children demonstrated a markedly higher level of HbO_2_ saturation in the right frontal pole during Go/No-Go tasks after undergoing physical activity intervention. This finding corroborates Damrongthai et al.’s (2021) observation that acute physical activity significantly increased HbO_2_ saturation in the PFC, including the frontal pole, during Stroop task performance (Damrongthai et al., 2021). The frontal pole is considered a core region supporting cognitive activities, exhibiting hemodynamic changes across tasks ranging from simple conditioned response paradigms to complex tests involving memory, judgment, or problem-solving (Burgess et al., 2007). Byun et al. demonstrated that acute physical activity improves EF through heightened arousal levels and increased neural activation in the DLPFC and FPC (Byun et al., 2014). Post-exercise activation in the frontal pole may reflect ascending projections from the reticular activating system to the PFC, suggesting acute physical activity could induce elevated oxygenated hemoglobin concentrations in the frontal pole and alter the activation patterns of multiple neuromodulatory systems (Dietrich & Audiffren, 2011). The significant increase in oxygenated hemoglobin concentration observed in the frontal pole after acute physical activity in this study may result from heightened arousal levels. This elevation prompts the release of neurotransmitters from neuromodulatory systems projecting to the PFC, thereby activating the frontal pole and improving individuals’ IC capabilities.

Following cognitively engaging physical activity interventions, participants demonstrated a significant improvement in Go/No-Go task accuracy, with correct reaction times significantly lower than pre-intervention levels, aligning with prior research (Kolovelonis et al., 2022a). This reduction may be attributed to the incorporation of situational interference, mental control, and exploratory discovery within cognitive engagement physical activities, which altered children’s cognitive strategies (Best, 2010; Ferreira Vorkapic et al., 2021). The Dual Mechanisms of Control (DMC) theory posits two modes of cognitive control: proactive control and reactive control. Proactive control facilitates goal achievement by selectively focusing on pertinent cue information at the outset of a task and perpetually modifying plans and behaviors (Braver, 2012). Physical activity levels correlate positively with proactive control; individuals with higher activity levels demonstrate superior top-down attention allocation and greater efficiency in sustaining goals (Ren et al., 2023). Studies show that short bursts of physical activity that require coordination and thinking greatly improve children’s reaction times and performance on selective attention tests (Kulinna et al., 2018). Consequently, acute physical activity interventions that include cognitive engagement elements produce immediate enhancements in inhibitory control. This effect presumably occurs when people perform physical activity in situations requiring coordination and cognitive effort, necessitating elevated levels of selective attention to consistently monitor task and environmental fluctuations. This encourages children to employ more proactive cognitive control strategies to prepare for and respond to cognitive tasks. However, this study only observed a significant improvement in accuracy in the cognitively engaging physical activity group during simple effect analysis, with correct reaction times lower than the conventional physical activity group in the post-test. Given the non-significant interaction effect, this result requires very cautious interpretation and warrants validation through subsequent studies with larger samples and more challenging tasks.

The CEPA exhibited a significant increase in L-DLPFC HbO_2_ concentration during Go/No-Go task execution, consistent with previous research (Ji et al., 2019). Cognitive control theory posits that cognitive regulation comprises two essential components: one responsible for executing control and another for monitoring task performance and signaling when control adjustments are needed (MacDonald et al., 2000). The DLPFC mediates top-down control by maintaining attentional set (Vanderhasselt et al., 2006). Previous studies have confirmed that transcranial direct current stimulation targeting the DLPFC produces significant improvements in EF with pronounced left-hemisphere lateralization. These behavioral gains are accompanied by distinct physiological changes, including reduced N200 amplitude and increased P300 amplitude (Dubreuil-Vall et al., 2019). Li et al. observed differences in PFC HbO_2_ concentration during Stroop tasks between long-term open-motor skill and closed-motor skill practitioners. They found significantly elevated blood oxygen levels in the DLPFC and frontal pole of the open-motor skill group, suggesting novel, variable tasks and environments may be key factors in enhancing practitioners’ neurocognitive function (Q. Li et al., 2024) Furthermore, compared to low-cognitive-engagement physical activity, subjects exhibited shorter N2 latency after a 20 min high-cognitive-engagement physical activity intervention, significantly enhancing the speed and efficiency of neural processing when confronting conflicts (Chueh, 2023). Thus, research indicates that compared to conventional physical activity, CEPA provides children with richer cognitive stimulation, additionally activating the L-DLPFC. This facilitates the maintenance of attentional set, thereby improving IC abilities.

### 4.2. The Immediate Effects of CEPA on Working Memory and Its Neural Mechanisms

The results of this study indicate that following an acute physical activity intervention, only the cognitively engaging physical activity group demonstrated a significant reduction in reaction time post-intervention, while the conventional physical activity group showed no significant changes before and after the intervention. This suggests that during acute physical activity, increasing cognitive load in both task and environmental aspects can provide additional cognitive stimulation to preschool children’s working memory. This may help individuals mobilize cognitive and motor resources for subsequent working memory tasks, thereby enhancing processing efficiency in working memory tasks among preschool children. O’Brien et al. (2021) found that compared to closed motor skills, children’s WM task scores significantly improved after a single open motor skill intervention, consistent with the present findings. Meanwhile, in an acute physical activity study, Preston et al. (2025) randomly assigned preschool children aged 4–5 years to groups characterized by different levels of physical activity (high/low) and cognitive engagement (high/low). Results indicated that only when both physical activity and cognitive engagement were high did participants demonstrate significantly superior working memory performance post-intervention compared to other groups. This finding relates not only to the potential developmental sensitivity of working memory in this age group, making them more responsive to such interventions, but also to the heightened demands on visuospatial information processing imposed by task and environmental adaptation processes. The cognitive engagement hypothesis asserts that the cognitive advantages of physical activity arise not solely from physiological alterations but also from the cognitive exertion and intrinsic cognitive requirements necessary for the performance of intricate motor tasks (Pesce, 2012). Compared to conventional physical activities, cognitive engagement physical activities offer age-appropriate novelty and challenge, effectively sustaining young children’s interest in learning and thereby promoting the development of related cognitive abilities (Tomporowski et al., 2015). Additionally, the significant improvement in WM capacity among preschool children in this study may stem from the appropriateness of the WM measurement tools. Baddeley’s working memory model has two parts that are especially useful for measuring young children’s working memory: the verbal loop and the visuospatial sketchpad (Baddeley, 2000). Some studies use cognitive tasks that test WM and use the verbal loop (Jäger et al., 2014; Miyake et al., 2000), while others look at how information is processed in relation to visual and spatial relationships (Zimmermann et al., 2021). Given that preschool-aged children tend to process visual information through visual representations rather than verbal encoding and repetition (Hitch et al., 1989), the animal picture 1-back cognitive task employed in this study avoids language-specific constraints. By leveraging the visuospatial component of WM (Lin, 2012), this task may enhance children’s sensitivity to the 1-back task, potentially leading to the observation of significant differences in WM outcomes.

Compared with the conventional physical activity group, the cognitively engaging physical activity group exhibited significantly elevated bilateral DLPFC HbO_2_ concentrations during the 1-back WM task, consistent with previous findings. Li et al. demonstrated that following acute physical activity of identical intensity, the closed-skill group—requiring motor memory and higher cognitive load—exhibited significantly greater PFC HbO_2_ concentration during the WM task compared to the open-skill group (Q. Li et al., 2024). As a core hub for monitoring and manipulating WM, the DLPFC shows widespread activation across various WM tasks (Barbey et al., 2013). The MEM model suggests that when cognitive demands are low, reflective processes dominated by the right PFC suffice for task completion; as task complexity increases, additional involvement of the left PFC may provide compensatory support (Balconi, 2013). Consistent with this, Webler et al. (2022) demonstrated that high cognitive load, compared to low load, effectively enhances activation in the prefrontal-parietal network and the cingulate-insular cortex region while inhibiting the default mode network. In this study, the bilateral dorsolateral prefrontal activation observed after cognitively engaged physical activity validates arousal theory: physical activities requiring higher cognitive engagement activate brain regions involved in EF, enhance physiological arousal, induce more specific cognitive activation, and thereby further strengthen WM (Best, 2010). Therefore, research indicates that acute cognitively engaging physical activity can immediately enhance working memory in preschool children, with the improvement correlated to changes in oxygenated hemoglobin concentration in the bilateral DLPFC. However, whether the promotional effect of single-session cognitive engagement during physical activity on working memory follows an inverted U-shaped pattern remains to be directly tested in studies systematically manipulating cognitive engagement arousal levels.

### 4.3. Immediate Effects of CEPA on Cognitive Flexibility and Its Neural Mechanisms

The results of this study suggest that acute physical activity interventions, regardless of whether they are combined with cognitive engagement, did not produce significant differences in behavioral performance on the DCCS task or in related PFC HbO_2_ levels. This finding corresponds with the conclusions of previous studies. Preston et al. demonstrated that acute physical activity under various conditions did not produce significant immediate improvements in CF among preschool children (Preston et al., 2025). EF is not a single construct but comprises multiple interconnected yet separable subcomponents, developing according to a hierarchical sequence. Models of EF development indicate that IC and WM serve as foundational building blocks upon which CF develops (Diamond, 2013). Another study indicates that the preschool years represent a critical period for rapid enhancement of IC and WM abilities, whereas the maturation trajectory of neural circuits underlying CF lags behind, typically reaching maturity in late childhood or adolescence (Garon et al., 2008). Similarly, a meta-analysis indicates that acute physical activity exerts differential effects on EF subcomponents, with smaller effect sizes observed for CF compared to WM (Liu et al., 2020). Consequently, preschool children demonstrate significant improvements only in IC and WM following acute physical activity interventions, likely due to developmental stage differences across EF subcomponents.

## 5. Conclusions and Recommendations

Acute cognitively engaging physical activity can provide immediate impacts on oxygenated hemoglobin concentration in the prefrontal cortex of preschool children, augment working memory ability, and offer possible benefits in enhancing inhibitory control. The fundamental mechanisms may be intricately linked to alterations in cognitive strategies, prolonged interest, and physiological arousal elicited by cognitive engagement during stimulation. This study provides scientific evidence for applying cognitive engagement elements in cognitive development and theoretical support for designing targeted physical activity intervention programs.

Limitations and Future Directions: (1) Regarding participant selection, the study focused on the effects of cognitively demanding physical activities on executive function in preschoolers aged 5 to 6 years. The study lacked external comparisons with preschoolers aged 3 to 5 and internal studies of gender differences, failing to determine whether the effects of cognitively demanding physical activities on preschoolers differ by age or gender. Future research may encompass children of varying ages and genders as subjects to better investigate the attributes of how physical activity enhances executive function development in this demographic. The limited sample size of this study constrains the interpretative validity of the findings, indicating the need for larger samples in subsequent research. (2) In experimental design, the study examined the effect mechanism of acute cognitively engaging physical activity on preschool children’s executive function. Future research could further investigate the duration of this effect, as well as the impact and underlying mechanisms of long-term cognitive engagement in physical activity on preschool children’s EF. (3) For data analysis, the study used fNIRS technology to look at how the concentration of oxygenated hemoglobin changed in areas of interest. Future research could explore functional connectivity between different brain regions to further investigate the underlying neural mechanisms through which cognitive engagement in physical activity enhances EF.

## Figures and Tables

**Table 1:** Experimental intervention content table.

Motor Skill Type	CPA	CEPA	Cognitive Engagement Element
Balance and stability motor skill	Single/Double-Leg Static Stance	Animal Imitation: Reproduce various animal stances through bodily gestures.	discovery
	Balance Beam Walking	Rainbow Plank Bridge: Identify the balance beams of various colors and proceed across them in a designated sequence.	mental control
	Rolling Forward on a Triangular Mat	Compass Roll: Execute a roll in a designated direction.	situational interference
Operational motor skill	Straight-Line Dribbling	Soccer Star: Dribble around the obstacles.	situational interference
	Long-Distance Sandbag Toss	Go Game Toss: Throw a sandbag into a designated agility circle from a distance.	mental control
Mobility motor skill	Sideways Walking	Crab Walk: Move sideways by stepping over and crawling under obstacles to reach the finish line.	mental control
	Circle Field Run	Red Light Stop, Green Light Proceed: Move in accordance with the instructor’s colored signals. (Green: normal running; Red: stop; Yellow: slow down).	situational interference
	Forward Frog Jumps	Spatial Frog Jumps: Jump between lotus leaves at different distances.	mental control

Note: CEPA = Cognitively engaging physical activity; CPA = Conventional physical activity.

**Table 2:** Channels corresponding to the location information of brain regions of interest.

Prefrontal Cortex Region	Channel
right-dorsolateral cortex (R-DLPFC)	6, 7, 10, 11, 15, 16, 19
left-dorsolateral cortex (L-DLPFC)	8, 9, 13, 14, 17, 18, 23
right-frontopolar (R-FPA)	20, 24, 25
left-frontopolar (L-FPA)	22, 26, 27

**Table 3:** Demographic characteristics of subjects.

Indicator	CEPA	CPA	*t* Value	*p* Value
Gender (Male/Female)	15/12	14/15		
Age (years)	5.19 ± 0.38	5.16 ± 0.29	0.254	0.803
Height (cm)	112.11 ± 4.15	113.21 ± 3.75	−0.890	0.385
Weight (kg)	19.34 ± 2.44	19.46 ± 1.68	−0.146	0.885
Body Mass Index (kg·m^−2^)	15.35 ± 1.35	15.21 ± 1.51	0.256	0.801
Basic Motor Skills	8.64 ± 2.74	7.39 ± 2.25	1.903	0.062

Note: CEPA = Cognitively engaging physical activity; CPA = Conventional physical activity.

**Table 4:** Average heart rate during exercise (M ± SD).

Indicator	CEPA	CPA	*t*-Value	*p*-Value
Heart rate (beats·min^−1^)	161.91 ± 17.095	158.24 ± 17.212	1.720	0.087

Note: CEPA = Cognitively engaging physical activity; CPA = Conventional physical activity.

**Table 5:** Relationship between HbO_2_ concentration changes and Go/No-Go ACC and correct RT variation.

	R-DLPFC	L-DLPFC	R-FPA	L-FPA	Go/No-Go ACC
L-DLPFC	0.652 **				
R-FPA	0.521 **	0.318 *			
L-FPA	0.429 **	0.469 **	0.554 **		
Go/No-Go ACC	0.053	0.077	0.022	0.055	
Go/No-Go RT	−0.131	−0.501 **	−0.153	−0.411 **	0.067

Note: * *p* < 0.05, ** *p* < 0.01; ACC = Accuracy; RT = Reaction Time.

**Table 6:** Relationship between HbO_2_ concentration changes and 1-back ACC and correct RT variation.

	R-DLPFC	L-DLPFC	R-FPA	L-FPA	1-back ACC
L-DLPFC	0.579 **				
R-FPA	0.421 **	0.318 *			
L-FPA	0.290 *	0.343 **	0.576 **		
1-back ACC	0.116	0.114	0.088	0.184	
1-back RT	−0.139	−0.299 *	−0.278 *	−0.249	−0.125

Note: * *p* < 0.05, ** *p* < 0.01; ACC = Accuracy; RT = Reaction Time.

## Data Availability

The datasets generated and analyzed during the current study are not publicly available due to privacy protection for the participants but are available from the corresponding author upon reasonable request.

## Abbreviations

The following abbreviations are used in this manuscript:

CEPA	Cognitively engaging physical activity
EF	Executive function
fNIRS	Functional near-infrared spectroscopy
IC	Inhibitory control
WM	Working memory
CF	Cognitive flexibility
FPC	Frontopolar Cortex
DLPFC	Dorsolateral Prefrontal Cortex
HbR	Deoxyhemoglobin
HbO_2_	Oxyhemoglobin
PFC	Prefrontal Cortex
DCCS	Dimensional Change Card Sorting
